# “Neither of meate nor drinke, but what the Doctor alloweth”: Medicine amidst War and Commerce in Eighteenth-Century Madras

**DOI:** 10.1353/bhm.2006.0009

**Published:** 2006

**Authors:** Pratik Chakrabarti

**Keywords:** Madras, “black doctor,”, surgeon, Country trade, bazaar, Tanjore pills, Coromandel, hospital, English East India Company

## Abstract

Madras in the eighteenth century was a site of continuous warfare sparked mostly by trading interests. This paper studies how these influences of hostility and commerce shaped the medical establishment of the English East India Company. It begins by analyzing the struggle of the medical establishment to cope with military and logistical requirements; it then shows how the Coromandel trade provided a peculiar dynamic to the practice of medicine in Madras. By aligning the history of medicine with that of trade, the paper traces the parallel trajectories of intellectual and material wealth. The development of modern medicine is seen as a process of adjusting to and engaging with diverse ideas and items—sometimes co-opting them, sometimes realigning them in new modes of production.

The port of Madras (Chennai) on the Coromandel Coast of India was founded in 1639, and Fort St. George in the 1640s, by the English East India Company (EEIC, 1600–1858), a joint-stock corporation of English merchants, as part of their network of warehouses or “factories” protected by military settlements and forts across South and East Asia. Throughout[Other P-1] the eighteenth century, Madras was a site of continuous warfare sparked mostly by trading and territorial interest. The battes were with the French (the First and Second Carnatic Wars, 1744–48 and 1749–54) as well as with the rulers of Mysore, Hyder Ali, and Tipu Sultan (the four Mysore Wars of 1767–69, 1780–84, 1790–92, and 1799). As a direct consequence of these conficts, the establishment of medical services soon followed the setting up of the town and the fort, formally coming into existence with the founding of the hospital in 1664.

Historians of the emergence of medical institutions and hospitals, particularly those of the EEIC, from the late seventeenth century have mainly focused on the theme of war, sometimes characterizing the medical practices as “military medicine.”^1^ I will suggest in this paper that military medicine in Madras was not only about survival in the battlefield: it was also shaped by the material culture of trade and the politicai appropriation subsequent to the military campaigns that unfolded in this region over the eighteenth century. I will address some of the key issues in our growing recognition of the role of the empire in shaping European scientific and medical knowledge,^2^ and I will highlight the need to understand the flow of ideas in terms of material and political shifts, particularly in the colonies.

In the first part of the paper I discuss how the medical establishment was created by the nature of the EEIC's settlement and military activities in Madras. The next section demonstrates how commerce, the chief concern of the EEIC, was also crucial to the growth of that establishment. The practice of medicine in Madras needs to be mapped along the trade routes, in the items of barter and in the markets of exchange, to construct a history of treatment enriched by trade.

Finally, I will show that medicine in Madras has to be understood in terms of the shifting political, military, economic, and intellectual dynamics on the Coromandel Coast. At a more general level I will show how modern medicine developed as an attempt to adjust to and engage with the multifarious collections of ideas and items—sometimes co-opting them, sometimes realigning them in new modes of production. Madras[Other P-2] at the confluence of war and trade, of ideas and ingredients, provides an ideal setting for such a study.

## War and Medicine in Madras

C. A. Bayly has suggested that the financial and military momentum of the EEIC's army was the mainspring of British expansion, particularly in India, during the early eighteenth century.^3^ The establishment and growth of the military medical department in Madras Presidency needs to be seen as a product of that growing momentum.

An early concern of the EEIC was to ensure the health and discipline of its European soldiers and seamen at its foreign settlements. In 1664 William Gyford and Jeremy Sambrooke wrote to the governor of Madras, Sir Edward Winter, saying that a hospital and regimented health care were necessary to treat the English soldiers:

soe the fresh souldiers which came forth this year takeing up their habitation in the bleake winde in the hall, fell sick. Four of them are dead: about tenn remaine at this time very sick and complaine (and it seems not without Reason) that the wages are not suffcient to supply them with the necessary now in this time of their sickness. Soe, Rather than see Englishmen dropp away like doggs in that Manner for want of Christian Charity towards them, we have thought it very Convenient that they might have an house on purpose for them, and people appointed to looke after them and to see that nothing comes in to them, neither of meate nor drinke, but what the Doctor alloweth.^4^

Thus the earliest hospital in Fort St. George was established on 16 November 1664 with John Clarke as the first surgeon. In 1688, for want of space, it was moved to the northern end of the barracks.^5^

As pointed out by Mark Harrison, it was only in the mid-eighteenth century that the expansionist policy and “open warfare” of the Company led to the employment of regular forces in Bengal and Madras Presidencies.^6^ Until then the hospital in Madras remained within the fort and served only the European military and civilian population within it. The[Other P-3] wars with the French had brought unprecedented numbers of European troops, stores, and artillery to India.^7^ In the middle of the Second Carnatic War, in 1750, Commander-in-Chief Bascowen took possession of the hospital to serve as barracks in the fort. Surgeon Andrew Munro subsequently wrote to the Madras Military Board that the hospital site had become rather “crowded” and the army had taken up the apartments of the mates.^8^ By this time new expansion was taking place outside the White Town (demarcated for the European population), for the population had outgrown it and the fort area lacked adequate housing. By the 1750s the original Indian settlement in the Black Town was demolished to expand the buffer zone around the fort. The Indian population was now gathered within the suburban quarters of Muthialpet and Peddanaikpet, the “new” Black Town.^9^Peddanaikpet was the area where most of the Vel-lalar groups, including the EEIC's *dubashes,*^10^ were settled,^11^ and it was to Peddanaikpet that in 1752 the hospital was ordered to be shifted outside the fort for the first time.^12^

Subsequent wars also created the need for enlargement of the hospital facilities. In November 1758 the surgeons Robert Turing, James Wilson, and T. S. Hancock sent a comprehensive proposal to George Pigot, president and governor in council of Fort St. George, for a new hospital—the[Other P-4] first outline of a modern hospital in Madras. The new hospital was to be divided into separate wards for patients suffering from fevers, fluxes, venereal diseases, malignant fevers, and smallpox, and for invalids and “Incurables”; there was also to be a room each for operations and the dispensary. Every patient was to be examined by the surgeon or one of the assistants upon admission.^13^ The diets of the patients were also specifed.^14^ Assistant surgeons were to help the principal surgeon in performing operations. One of the assistants was also to act as the apothecary, prepare and distribute medicines, give injections, and salivate^15^ patients with the help of two Indian *coolies*^16^ who were also to act as cooks. The Indian under-assistants and the *dubashes* were to make and prepare dressings as well as to help in preparing medicines by pounding the mortars. Local women were to clean the wards and provide the hospital with water, and a “head coolie” was to supervise the Indian subordinates.^17^

The Military Board initially approved most of the submissions, but war caused these plans to be shelved. In May 1771 the Board finally resolved to accept the proposals for the construction of a new hospital building, following closely the administrative and architectural outlines of the 1758 plan.^18^ In 1772, the present-day double-block buildings of the Madras General Hospital were inaugurated. Today the hospital stands in central Chennai, across the road from the headquarters of the Southern Railway.

The practice of medicine in Madras also had to engage with battlefeld and logistic concerns. Victory did not ensure well-being in a hostile land. In January 1760 the English recorded one of their most decisive victories in Deccan, at Wandiwash against General Lally's French troops. The victorious General Eyre Coote, however, found the battle scenes “dreadful” due to the lack of medical support in the field:

Such a multitude of poor objects, and not in my power to give them the least assistance for want of every one necessary requisite for an hospital. I make no doubt upon this representation you will do everything humanity can direct. If it is possible to send surgeons and proper people from Madras to attend the wounded here who are very numerous, you may by that means save the[Other P-5] lives of many gallant men, several of whom have not been dressed since the day of action.^19^

The lack of either adequate flying field hospitals or mobile staff in India made the transportation of medicines from the hospitals to the battlefields, and of the sick or wounded from the battlefelds to the hospitals, a major problem. The situation was made worse by the favorite tactic of both Hyder Ali and Tipu Sultan of starving out the British armies by cutting off their supplies.^20^

Senior Surgeon Colley Lucas complained about the lack of coolies to carry the sick in the field.^21^ The other problem that continuously troubled the medical establishment was that of the *doolies:*^22^ In 1788 surgeon Maxwell Thompson wrote to the Hospital Board (also referred to as the Madras Medical Board, established on 14 April 1786) in Madras that for the want of *doolies* the sick had to walk to the hospital, a distance of two to three miles, under the hot sun; this problem of logistics was also related to the confusions regarding command and authority faced by English surgeons in Madras, for he added: “I also request to know if these Dooley boys are to be under my immediate direction, the difficulty of getting them when wanted, having often occurred from the Dooley boys believing that they are to obey no orders given them but from the person who pays them.”^23^Throughout the later decades of the eighteenth century the Madras Medical Board continued to experiment with new designs for *doolies* and bullock-drawn carts to carry the sick and the wounded.^24^

The supply of medicines was the main concern in the field and the provincial hospitals. The main provision for the Madras hospital came in the form of medicine chests from Europe; these were often destroyed or lost on the long voyages from Europe, and the surgeons then had to re-[Other P-6] sort to the private collections of the Europeans.^25^ In 1722, when the ship *Nightingale* with the annual supplies of medicine for Madras was lost at sea, crisis struck the hospital: the president wrote to the Board that the surgeons had frequently told him “of the want of Medicines in the Hospital occasioned by the loss of the Nightingale,… It is therefore Agreed that the same be purchased allowing twenty five per cent advance and that the amount thereof be paid for out of Cash.”^26^ There were several such occasions.^27^

Complaints were also made about the poor packaging of medicines from Europe. In 1774 Gilbert Pasley and James Anderson wrote to the Board about the European medicines received for 1773, which had arrived “much damaged on account of the careless and injudicious Package of them,” and they added that the “supply of surgical Instruments is likewise greatly deficient.”^28^ In 1787 the Hospital Board wrote to the government about an “Evil”—the poor condition and quality of medicines sent from England:

The Hospital Board beg leave to take this opportunity of informing Government of the imperfection and indeed the inutility of several of the Medicines brought from Europe, especially Cantharides or blistering flies, the efficacy of which is so considerable in many critical disorders, and the consequent disappointment so great.^29^

The situation was particularly grave in the provinces of the Presidency, which depended on Madras for their supply of European medicine. In 1787 John Duncan, assistant surgeon with the Battalion of Native Infantry, represented the “great want” of a medicine chest to carry medicine easily and to protect it from pests.^30^ Pasley and Anderson described the “indigenous” modes used for transporting the medicines to the provinces:[Other P-7] “the annual supply is divided, parcelled out and packed in such awkward utensils as the Country furnishes, put into wicker baskets and sent on the head of Cooleys along the Coast almost from Cape Comorin to Balisore [Balasore]…. many are lost by carelessness, in Rivers, Monsoon, and by Enemies.”^31^ In 1792 George Binney wrote from Machilipatnam that most of the European medicines sent from Madras were damaged and fresh supplies were needed.^32^

But the “field” of medical practice and treatment in the Madras Presidency stretched beyond the zones of military confict and bloodshed, often to the “Country” (in its Anglo-Indian sense), which was also the source of supply for medicines. This is where the story of medicine at Madras is linked to the main concern of the EEIC, its trade in the Indian Ocean world.

## Medicine in the Marketplace

The practice of medicine beyond Europe in the eighteenth century posed many challenges for the Europeans. Apart from war, the climate and the customs of the Coromandel Coast also troubled the European constitution and conscience. Charles Curtis, a surgeon from England who briefy visited the hospital during 1782–83, found it and its surroundings rather inhospitable:

The country round was more than once overrun, and plundered by the horse troops of Hyder Ally. So that our supply, even of fresh meat, and much more of vegetables and fruits, was by far too scanty. And a few imperfectly cultivated and unripened fruits, with the cocoa-nut juice called *toady,* was all we could procure for our scorbutics. Another source of unhealthiness arises from the situation of the town, and fort of Madras itself, built upon a low, flat and sandy beach, and surrounded almost entirely by stagnant swamps, or ponds of stagnant water, which are not only a receptacle for every sort of corruption and filth, but have their edges, as well as the beach on the other side, covered at all hours of the day with the natives, in the act of relieving nature from their burdens, to take advantage of the water for washing afterwards, which is their invariable custom. What a source of putrid exhalations under a vertical sun!^33^

But where there was grime, there was also bounty. Throughout the years of war the EEIC was establishing itself as the principal trading power in these parts. Madras, as its stronghold in this expansion, was the hub of various networks and arbitrations and, as suggested by some historians, a site not of degeneration but of “vitality.”^34^This meant that the surgeons of Madras were placed in an interesting confluence of ideas and ingredients, and consequently, their intellectual lives were eclectic. Samuel Brown, a surgeon in the hospital in the late seventeenth century, spent most of his time collecting medicinal plants from the neighboring forests and discussing their virtues with the “locals.” Brown had been engaged by the London apothecary James Petiver to dispatch dried plants and seeds to England; Petiver then analyzed them in relation to similar collections made in England and elsewhere, and published them in the *Transactions* of the Royal Society.^35^ Edward Buckley, chief surgeon at Fort St. George in the same period, sent to Hans Sloane at the Royal Society a “China Cabinet” full of instruments and simples used by Chinese surgeons.^36^ Paul Jodrell, physician to the nawab of Arcot, discovered a “Lusus Naturae,” a boy named Peripa with physical deformities, whom he kept in his garden for observation, and he planned to send sketches of Peripa to the Royal Society.^37^ Jodrell also met “Mimood Saib,” an alchemist who claimed to convert metal into gold; he tried to convert the alchemist to his logic of science, but was obliged to leave him “obstinate in his Opinion.”^38^ James Anderson, the surgeon general at Madras, obtained a piece of land near Fort St. George where he cultivated and experimented with cochineals; in 1787, his friend and namesake James Anderson (LL.D.) laid before[Other P-9] the Royal Society of Edinburgh a specimen of a new species of cochineal lately discovered on the coast of Coromandel.^39^

The EEIC itself was deeply entrenched in the social life of Madras and its surroundings in the early decades of the eighteenth century. The local nayak or the nawab of Carnatic had little visible control over Madras, a port city established by the British. As Holden Furber described it: “Fort St. George was much more impressive than Fort William [at Calcutta]. The Governor and Council actually ruled over a sizable local population, composing caste disputes and making authority felt well outside the so-called ‘white’ and ‘black’ towns of Madras, especially in the countryside where the weavers of the ‘Company's cloths’ lived.”^40^

While problems of infrastructure and logistics plagued the medical establishment of Madras, surgeons from very early on had discovered other sources for the supply of medicines. The eighteenth-century history of the hospital shows several instances where surgeons depended on the local bazaars. By the middle of the century, Country medicines or bazaar medicines, as they were referred to, had become a regular part of the supply for the establishment and the surgeon general's records maintained separate requisitions for them. One of the main proposals of the 1758 report from the surgeons was to have separate rooms for European and Country medicines.^41^ In 1786, surgeon James Whyte from Tanjavur (Tanjore in Company records) asked the governor for permission to buy medicines from the bazaars, instead of the supplies from Madras.^42^In 1789 the annual cost of bazaar medicines supplied by the storekeeper for the Native Corps alone was more than 769 pagodas.^43^

The surgeons' conversance with local supplies is evident in the 1762 proposal of the surgeon James Wilson to personally supply the hospital with cots, clothing, Country medicines, servants, and all other necessities except “Europe Medicine”; he also proposed to hire a *dubash* for his shop to assist in the preparation of medicines and to make up dsressings, [Other P-10]and coolies to perform menial and domestic tasks.^44^ In 1792 the Hospital Board submitted to the government separate plans for each hospital for supplying bazaar medicines.^45^ It also appointed Indian contractors for providing Country and bazaar medicines at Madras and the provincial hospitals.^46^

Private procurement, however, continued. In 1796 the surgeon Bernard McMahon wrote from Ceylon that he had bought medicines from the bazaar to treat his patients suffering from ulcerous putrefaction.^47^ In 1792 W. M. Kellop, surgeon, Artillery, wrote to Terence Gahagan, head surgeon of Vellore, stating that because the supplies received from Madras were “very inadequate” he had to purchase bazaar medicines with his own money.^48^ Purchase orders for bazaar medicines were regularly sent by the provincial surgeons to the Hospital Board.^49^ In 1802 G. Dunbar wrote from Ganjam about intermittent fevers, which were successfully treated by a locally acquired substitute for the Peruvian bark that he called the “Hindustanee Fever Pill.”^50^

One reason for the growing need for bazaar medicines was the increasing numbers of Indians recruited in the forces, who were generally treated with indigenous medicines. During 1765 a major expansion of the Native Infantry took place, and at about the same time the government sanctioned the appointment of a “Native” doctor to each sepoy battalion with the pay of a havildar.^51^ The surgeon general's records maintained annual lists of bazaar medicines supplied by the storekeeper for the use of the “Native Corps.”^52^ This increase was truly a case of “native” medicines for “native” bodies.

What constituted this “Country medicine”? At one level, it can be defined in terms of supply: anything that was procured locally and was not sent from Europe constituted “Country medicine.” Behind “Country medicine” were the local “native” practitioners and informants on whose[Other P-11] knowledge the surgeons were dependent both for procuring the suitable ingredients and for their preparation and application. We now need to understand the nature of this European involvement in local medicines and practices.

## Country Trade, Country Medicine

“Country trade” was the term used by the Europeans for the local trade of South and Southeast Asia in which they participated increasingly, often privately, throughout the eighteenth century. One of the important successes of the European trading powers was their ability to divert this trade not only to the ports controlled by them but also to the goods of their interest.

During the eighteenth century the history of the hospital in Madras was shaped as much by European trade to and from the Coromandel Coast as by the requirements of the Carnatic and Mysore wars. In the early decades of the century doctors like Robert Turing were generally engaged in private trade.^53^ Until 1697 the medical offcers bought drugs from the bazaar for hospital use and submitted the bills at long intervals; in 1697, the Council stressed that the surgeons needed to submit such bills monthly.^54^ Trade was crucial to medicine in terms of proft as well. In his advertisement for Samuel Brown's Madras collections in the *Philosophical Transactions,* James Petiver referred to an earlier discovery of a root in the East which was found to be particularly useful in epileptic and convulsive diseases, justifying, according to Petiver, the need to “better understand” such medicines and “to have them in greater plenty, so that even the poorer sort of People may receive beneft by it at a much moderate price, and the Merchant receive advantage by trading in an new Commodity.”^55^

Science and trade were thus closely interlinked. The main Madras contact of William Roxburgh, an EEIC botanist posted at Samulcottah (or Samalkot) in Andhra Pradesh, two hundred miles north of Madras, was Andrew Ross, the most prominent trading agent in that city involved in country trade. Ross procured books in Madras to send them to Samulcottah for Roxburgh; Roxburgh sent Ross a breadfruit plant, to be [Other P-12] forwarded to St. Helena. The two men corresponded regularly regarding the experiments on the cultivation of cinnamon, tobacco, indigo, and teak at Roxburgh's station at Samulcottah. Ross played an active role in propagating within his own network the scientifc virtues of Roxburgh's discovery of the astringent bark *Swietenia febrifuga*^56^

But there were also other links, less obvious, between “Country trade” and “Country medicine.” The term “Country medicine” was used by Europeans for the *local* medicines procured and used in South Asia and the West Indies (the term “local” being the suggestive one). My suggestion here is not simply that Country trade amplifed the use of Country medicine. Rather, this comparison can be used as an analytical tool to understand how trade can be seen as a metaphor for medicine, particularly for this period; how it signifes European involvement in the “native” or the local; and how that shaped the identities and content of both. In the *Oxford English Dictionary,* the “Anglo-Indian” connotation of “Country” is defned as “Of or belonging to India (or other foreign country), as distinguished from European; native.”^57^ This particular meaning is derived from eighteenth-century texts like Alexander Hamilton's *New Account of the East Indies* (1727) on trade in the Indian Ocean region.^58^ The term “native” as commonly used by Europeans to describe the local or the indigenous population of non-European parts signifes a “rootedness”—as distinct from “European,” to which was attributed a certain mobile and universal quality. At one level similar to trade, medicine in these parts tended to have two distinct orientations: Country medicine, and European medicine. At another level, the “local” and the “metropolitan” were linked in the European use of these traditions and in the dispatching of “native” pills, plants, and texts to Europe either as novelties or for “analysis” by the scientifc community back home.^59^

The history of eighteenth-century Country trade is rather checkered. Even before the Europeans established themselves in these parts, there[Other P-13] were major trading networks connecting the Coromandel to the east with Bengal and Southeast Asia, including the Malay Archipelago, up to China, and to the west with Surat and the African and Arabian coasts. Samuel Brown's notes provide a rich testimony of Portuguese and other early European connections in bringing aromatic and medicinal plants to South India and beyond.^60^ The complex routes of travel of a tropical medicinal species to Europe can be traced in the journey of the Columbo (or kalumba) root. Initially introduced into Europe by the Portuguese in the sixteenth century, it gained large-scale acceptance within the European medical fraternity first through Thomas Percival's *Essays, Medical and Experimental.*^61^ Yet its geographical and botanical origins remained a mystery, largely due to the Portuguese desire to retain their monopoly. The root was in fact originally collected from the eastern tropical territories of Mozambique, where it was traditionally called “kalumb” and used for dysentery and other diseases; to conceal its source, the Portuguese carried it to India and shipped it from there to Europe.^62^

On the Coromandel Coast and in Bengal, Country trade became prevalent in the eighteenth century, increasingly mingled with the trade with Europe. According to Furber, it was the commercial revolution of the early decades of eighteenth century that stimulated the growth of European participation;^63^ the increased need for ammunition, copper, and saltpeter among the European powers further encouraged this traffic. The Europeans here interacted with local merchant communities like the Armenians and Chettiars in developing coastal trade between Madras and Bengal, leading ultimately to China. From the middle of the century the control of this trade was transferred to the free merchants, or individuals associated with agency houses. Another important aspect was the growing dominance of the British in the trade; by the 1770s their supremacy was well established.^64^ This trade, now conducted from European strongholds such as Madras, Pondicherry, Nagapatnam, and Machilipatnam, contributed toward strengthening the British control over the Coromandel Coast.[Other P-14] Although huge profts were being generated, the EEIC suffered financial difficulties during this period, since much of the trade was private.^65^

## Trade and Treatment

The medicines that the British surgeons found in the markets of South India did not come only from the neighboring forests: Country medicine was sometimes “Country trade” medicine as well. The products had a long and syncretic history of trade and network, often being brought over from other regions and used locally as medicinal ingredients. The Europeans similarly used them as ingredients for their own medicines, and also bought “pills,” barks, and roots commonly in use in the surrounding regions. The *Materia Medica of Hindoostan* published in 1813 by Whitelaw Ainslie (superintendent surgeon, Madras) describes the complex lineages of bazaar medicines, which were “either the produce of Hindoostan, or…brought to it from Asiatic countries, and are to be met with in the Bazars of populous Towns; including many Drugs of the Tamool, Arabian, and Persian Materia Medica.”^66^

Important sites of Country trade like Machilipatnam also became signifcant points of procuring Country medicine. In 1788 the surgeon George Binney wrote from Machilipatnam to James Anderson, mentioning that “Ginglee (Sesame) Oil” used in the composition of ointments could be found there, for sesame grew widely and the oil could be procured locally in a state fresher than that from the Presidency.^67^ He in fact suggested large-scale local acquisition of bazaar medicines such as musk, camphor, opium, and so forth:

I believe I might venture to say almost every article of country medicines is to be purchased in the Bazar here, and I may add at almost every station of my department north of the Kistna, of as good a quality, at as cheap a rate, and some of them cheaper and fresher than at Madras. … it seems an expedient highly necessary to be adopted; and will be calculated to do credit to practice, by securing the very important end—of everything being of the purest kind, & of the best quality.^68^

The government, too, had realized the need for local procurement of medicines. In 1789 the Court of Directors wrote from London that it[Other P-15] was not ready to provide the entire supply of medicines demanded by the Hospital Board, particularly the “great quantity” of quicksilver and mercurial preparations, which needed instead to be collected locally.^69^ In 1796 Dr. John Hunter,^70^ an advisor to the Court of Directors, instructed the Hospital Board from London to procure ingredients like opium and tincture of opium locally in India because they would be cheaper there. He also asked the surgeons to get rhubarb and other items from China and other parts of Asia, for they would be fresher and cheaper.^71^ The Madras government responded positively to these suggestions. Benjamin Heyne, who had studied Tamil medical texts, was made assistant surgeon to the Mysore Survey in 1800, after Mysore territories were annexed, and one of his responsibilities was to supply bazaar medicines to the Company's army.^72^ Similarly, when Francis Buchanan Hamilton was sent to the newly acquired territories of Mysore and Malabar in 1800, he was under specifc instructions to provide the administration with useful information on “those natural productions of the country, which are made use of in arts, manufactures, or medicine,” an account “of the prevailing winds, and the effects of the air in its various states of heat, and moisture, on the human body,” and an estimate of the “salubrity” of the country.^73^ John Forbes Royle set out to compile his *Manual of Materia Medica* because the Medical Board wanted to ascertain whether supplies from Europe could be replaced by “articles indigenous in the country, or by cultivating exotics in the most suitable climates of the plains and mountains of North India.”^74^ Royle collected several drugs from the bazaars, tracing them to the plants, animals, and countries from which they were derived.

The substances listed as “Bazaar Medicine” in the hospital records are recognizable as items of trade as much as of treatment, and they suggest[Other P-16] interesting journeys through different territories: quicksilver (mercury), opium, nitre, gum arabic (sub-Saharan *Acacia senegal* and *A. seyal,* leguminous trees from Egypt),^75^ spogel-seeds (isabgol, *Plantago ovata*), aloes (derived from species of two East Indian genera, *Aloexylon* and *Aquilaria*), asafoetida (called *hing* in the Malay language),^76^ liquorice root (derived from the sweet root of various species of *Glycyrrhiza,* mostly from the East, but also found in Spain and Italy), cinnamon, China-root (the rootstock of a species of *Smilax,* from south China and the East Indies),^77^ columba (from Mozambique), cloves (*Caryophyllus*aromatics, from the Moluccas),^78^ camphor (from *Camphora officinarum*, a tree indigenous to Java, Sumatra, Japan, etc., and from other lauraceous trees, brought to India from China by Country trade ships),^79^ cumin seeds, dragon's blood (from a kind of cane from Java and Surinam, *Daemomorops draco),^80^* fennel seeds, gamboge (a gum resin obtained from various trees of the genus *Garcinia,* natives of Cambodia, Thailand, etc., used as a drastic purgative),^81^ ginger, gentian root (from China), gingilee oil (sesame oil, *Sesamum indicum,* from India),^82^ benzoin (a dry and brittle resinous substance, with a fragrant odor and slightly aromatic taste, obtained from *Styrax benzoin,* a tree of Sumatra and Java),^83^ mustard, oil of cinnamon (a common item of trade and treatment in the Dutch trade from Ceylon),^84^ radix (root) rhubarb (according to Ainslie, not found in India in great quantity, which was quite strange since the local practitioners used it so regularly; it could, however, be brought “with so little trouble” from China),^85^jalap, calamus[Other P-17] aromatics (from Malabar),^86^ nutmeg, mace (an aromatic spice consisting of the fleshy aril or covering surrounding the seed in the fruit of the nutmeg tree), galingale (the aromatic root of certain East Indian plants of the genera *Alpinia* and *Kæmpferia,* introduced to early medieval Europe by the Arabs),^87^ myrrh (a bitter, aromatic gum resin exuded by various Arabian and African trees of the genus *Commiphora,* in Abyssinia),^88^ and tamarind.^89^

Whitelaw Ainslie detailed the Tamil physicians' preparations of nitrous, muriatic, and vitriolic acids for medicinal purposes.^90^ He added that aloes, commonly found in the bazaars of India, was brought from China as well as Borneo, and that cardamom, commonly used as a medicine by English surgeons, grew on the Malabar Coast but was also brought from Gamboia (Cambodia).^91^ Tabasheer (an excretion of the joints of bamboo) was rarely found in India but was an important ingredient, and according to Patrick Russell it was brought to India as an article of trade.^92^ Tamarind, according to Ainslie, was considered by both Europeans and “native Indians” as cooling and laxative.^93^ Mercury, the vital medicine on the Coromandel Coast, was closely related to trade in these parts:

We are informed by Captain Turner, that, at Tessoolumbo in Thibet, Cinnabar is found which contains much Quicksilver: and I perceive by the little volume entitled “Remarks on the Husbandry and internal commerce of Bengal” that Mercury thus mineralized might be considered as one of the export articles of trade from Hindoostan; the greatest part of that valuable metal, however, which is exposed for sale in these provinces, is brought to us from China; where it is procured, both in its native purity (See Abbe Rochan's “Voyage to the Madagascar and the East Indies” p. 365–66) and combined with Sulphur.^94^

Thus while the bazaars were being seen as alternative sources of supply for medicines, European participation in trade and therapeutics had further syncretized these traditions. And Madras, the “open roadstead,”^95^ was often the site for this blend. We will next explore some of the consequences of these developments.

## The Madras Mortar

In July 1788 Mr. Strange, a surgeon near Tanjavur, wrote to Governor Archibald Campbell in Madras about a “native” practitioner, introduced to him by the Danish missionary Frederick Schwartz, who could prepare some kind of “snake pills” that apparently cured people bitten by snakes. Both Strange and Schwartz wanted this man and his pills to be examined by doctors in Madras. The governor asked for the man to be sent to Madras, where the Hospital Board was to make trials of the pills “for the purpose of having the merits of his discovery ascertained.”^96^

Analysis led to intervention. In September, William Duffn and other surgeons wrote to the Hospital Board that although the results of repeated trials were successful, some of the components raised questions, and they could “only recommend the Government to leave every practitioner to administer remedies, as his own judgement may direct as heretofore in cases of venomous bites.”^97^ They also extolled the Government's decision to publish the details of the ingredients since doubts regarding them remained among some medical experts.^98^

A simple adoption of these pills, which had promised a major cure, was not seen by the European medical establishment in Madras as an option, and analysis of the ingredients became vital to this European engagement with the Tanjore pills. In October 1788, after preliminary analysis, the Military Board approved and encouraged the use of the pills in cases of snake and mad dog bites.^99^ It stressed that the medical storekeepers should always maintain a supply of the pills.^100^ It also asked for a further and detailed analysis of the pills' ingredients to be advertised, along with their proper administration:[Other P-19]

that you will without loss of time draw up an accurate account of the names and proportions of Articles composing these Pills specifying in a particular manner the Malabar and English names of each Article, as well as the botanical names of such plants or Roots as are used in the Composition of these Pills and the manner of using the Pills. …The Board at the same time direct, that you will publish the same without delay in the Madras Courier, and that a quantity of these Pills may be immediately made up and sent to every Station under the Presidency.^101^

In November, James Anderson in his minute on the pills produced a detailed list of their ingredients and added that it was indeed arsenic that put their usage in doubt. He asked for a ban on the internal use of the pills, “wishing as I do to expel for the sake of humanity such noxious Drugs from our Materia Medica.”^102^ This view, however, was contested. William Duffn, Head Surgeon, Madras Hospital, in his Minute wrote that despite the arsenic he found the pills beneficial in most cases, and added that he had earlier transmitted the results to Patrick Russell, physician and naturalist to the EEIC, who had communicated them to the Royal Society, and Duffn was at present making experiments with the pills to corroborate the facts.^103^ Russell seemed to put an end to the debate: in his *Account of Indian Serpents Collected on the Coast of Coromandel* published in 1796 from England he described his experiments with these “Tanjore Pills” as inconclusive, and the pills were found to be rather ineffective.^104^

The blend of medicine and ideas in Madras was indeed intriguing. Thus sidelined in Europe, the pills and their ingredients had an interesting impact in India. In 1789 William Jones wrote to Anderson at Fort St. George, who seemed to have meanwhile changed his mind, about his own successes with arsenic:

I was much interested in your papers concerning the Hindu pills for the cure of persons bit by venomous snakes. Till I saw Duncan's Medical Commentaries, I had the same abhorrence with yourself to the use of arsenick in medicine, and had resolved to suppress a paper of a Native Physician concerning the cure of the Elephantiasis by small quantities of arsenick; but when I found that it had been safely given in England for intermittents, I ventured to print it in the second volume of our Transaction: … Poisons must, no doubt, be administered with extreme caution; but if we banish them from practice, what will become[Other P-20] of Antimony, Mercury & Opium, to say nothing of hemlock? And without the three first of them, what will become of mankind?^105^

But Russell's judgment largely put an end to the debate on the pills. Thus while spaces for new ideas and ingredients were being created in the peripheries, confrmation from the metropolis remained important. This duality becomes evident in Anderson's reply to Jones. Urging caution in the use of arsenic or any other poison in medicine, Anderson added:“[that] the natives of this country are better acquainted with the use of Simples than any of the Peoples may be readily admitted by all who consider the situation the stimulation of the country and the nature of their food and there is great probability that Chymistry has flourished here.”^106^

The “Tanjore pills” seem to have retained their ambiguous position for almost another century. In 1873, T. Lauder Brunton and J. Fayrer wrote that although they enjoyed a “large amount of popular confidence,” “when brought to the test of carefully conducted experiment, [they] failed, as might have been expected, to give any favourable result.”^107^ Interestingly, arsenic, the controversial ingredient, was one of the chief items sent by the Society of Apothecaries of London to India, St. Helena, Bencoolen (west coast of Sumatra), and Canton as medicine in the nineteenth century.^108^

It is worthwhile here to go back to Duffn's earlier defense of the pills and arsenic:

as we are still ignorant of the analysis of the exotic Plants, and other vegetable matter which are compounded with those minerals and how far they may counteract the Violence of the Arsenic we should not hastily condemn a Remedy where experience & occular *[sic]* demonstration have in many cases proved it may be given with safety, besides the internal use of arsenic is by no means[Other P-21] prohibited, it is given in cancerous cases and Intermittent Fevers by established Practitioners in Europe—My opinion is therefore that the formula should be published in the Courier.^109^

The conflict between the ocular and the analytical that is evident in Duffn's words in fact underlines a larger tension, both between different modes of scientifc credibility and between the metropolis and the periphery. This was manifested in the duality in the work of European scientists in India: the Madras hospital and its surgeons had continued to depend on the local bazaars and practitioners in this era of a distinct medical research and production in Europe. Ainslie began his *Materia Indica* with the common European reservation about the chemical skills of the local practitioners: “The preparations of mercury found in use amongst the Tamool practitioners give us but a poor opinion of their knowledge of chemistry”; but he then added: “Yet, after all, however much we may be inclined to smile at some of their strange mixtures, it must be confessed that the characterizing principles are generally correct, and that, every thing considered, there is, in the present state of knowledge amongst the *Vytians* and *Hakeems,*^110^ more to call forth our wonder than excite our contempt.”^111^ His footnote to the paragraph is even more interesting: “The Hindoos reckon mercury one of their most powerful medicines, but are very apt, *not always intentionally,* to induce by its use most frightful salivations.”^112^ Benjamin Heyne, while translating Tamil medical texts, remarked that, although many of the Indian practitioners were “quacks,”

the medical works of the Hindoos are neither to be regarded as miraculous productions of wisdom, nor as repositories of nonsense. Their practical principles, as far as I can judge, are very similar to our own; and even their theories may be reconciled with ours, if we make allowance for their ignorance of anatomy, and the imperfection of their physiological speculation.^113^

This is an important problematic of these interactions: they were as much to treat the sick in Madras as they were advertisements of peripheral research and novelties for Europe. The duality in the works of Ainslie,[Other P-22] Heyne, and Royle lies in their straddling of diverse worlds, in their divided loyalties, and in their narrations about Tamils, Siddha medicine,^114^ and Eastern bazaars for an essentially gentiemanly European scientifc audience.

The link between the local and the metropolitan is situated here. Signifcantly, Ainslie categorized his list of bazaar medicines as a part of “British Materia Medica” that “could be procured in the Bazars of Hindoostan,” and located them as much in the local markets as in the texts of Tamil scholars like Agashtier, *Agashtier Vytia Anyouroo.^115^* John Forbes Royle, born in Kanpur (in north India), entered the service of the EEIC as assistant surgeon and “made collection of all the drugs procurable in Indian bazaars, tracing them as much as possible to the plants, animals, and countries, whence they were derived”;^116^ he then went back to teach about these, among other things, at the King's College. Many of these accounts and encounters found mention in the *Edinburgh New Dispensatory*^117^ and the *Annals of Medicine.*^118^

These promotions and publications were informed by a search for legitimacy and autonomy by surgeons and physicians throughout the empire.^119^ In Madras, the surgeons were attempting to establish the city as an important site of medical research and treatment. The hospital had in fact emerged as a valuable training ground for young medical professionals: by 1772, it was training Europeans, Eurasians, and Tamils in allopathic methods of diagnosis and treatment, and the preparation of[Other P-23] medicines. These trained personnel were posted to various dispensaries in the district headquarters of the then Madras Presidency to assist the qualifed doctors.

In January 1775, Pasley and Anderson made an appeal that young medical assistants sent from England should possess certified qualifcations.^120^ However, they also explained the vital importance of training an assistant from Europe in Madras before putting him in full charge of a dispensary: “whatever knowledge or even practical abilities a surgeon may have learned in Europe, he is here a dangerous Member of Society until he has acquired by time and *experience* a local knowledge of the diseases *of this climate.”*^121^ An interesting incident ensued. Terence Gahagan, who had appeared for examination in Madras in October 1775, found the screening by Pasley too severe, and failed. He complained that he had been made “a Precedent in the cause of real examination.”^122^ The government gave him the alternative of returning to England, or of being tested by Anderson, Alexander Boswell, and Thomas Davies, and resolved “that all Surgeons' Assistant[s] in future be properly examined before they are admitted into the line of Surgeons.”^123^ Gahagan chose to return to England. The days of medical adventurers were pronounced to be over in Madras, as the hospital defined rigid criteria for inclusion within the establishment.

Pasley had played an important role in asserting the autonomy of medical research at the Madras hospital. Part of this independence was sought from the physicians at home. In 1771, when he was recommended by a London physician advising the Court of Directors to use castor oil in treating infammation of the liver, Pasley replied: “I am sorry to observe it as a proof of how ready Men of reputed eminence in Physic are to precipitate on the Work an opinion regarding the Virtues of particular Medicines, without a suffcient personal Experience of their effects; consequently the only conclusive Test, of the certainty of their powers and operations.”^124^ Pasley's defense of the Indian establishment was based on his argument that castor oil could work only as an evacuant; for liver infammation, which he found to be *“peculiar* to the Coast of Coromandel,” a “medicine of greater power” was needed.^125^ That medicine was[Other P-24] mercury. Pasley elaborated in another letter how the use of mercury in Madras for liver infammation was unique and needed an “experienced touch”; in India, the cause had to be looked for “beyond the intestinal canal. A deep-rooted obstruction generally supports the disease, in spite of unwearied evacuations.”^126^ The distinctive climatic, topographic, and cultural circumstances and the remoteness from metropolitan authorizing institutions and instructions had made familiarity and experience the crucial criteria for cognition and survival in Madras.^127^

Interestingly, Gahagan, who later joined the establishment, becoming first the chief surgeon at Vellore and then a member of the Medical Board, asserted the same sense of the novelty of medicine in Madras. In 1802, another centralization and militarization of the services was being undertaken. Gahagan, as the Second Member of the Medical Board, produced a note of dissent to a proposal by the Court of Directors to dispose of the General Hospitals of the Presidency in order to curtail costs. His primary defense of the hospitals was that they provided ideal training grounds for British surgeons in this country:

they open an ample field for young practitioners upon their arrival from Europe in this Country; They afford them an opportunity of acquiring a knowledge of local diseases and the best mode of treatment adopted in their care under the long experienced practitioners whence they may proceed better enabled to act of themselves, with confidence of success whenever their services may be required in detached situations.^128^

To Gahagan, Madras was the site where theory was put to test by experience, “for although a young man may come out well grounded in a theory, it still requires practice, experience and diligent observation to get[Other P-25] conformation of Medical Education.”^129^ He added that these hospitals had been integral to the history of the EEIC on the coast, fraught with wars and casualties: “their utility in every other respect, is manifestly obvious and has hitherto been experienced, on this Coast, which has been the principal scene of war for a series of years, and where the largest armies in India are generally assembled, and held in preparation upon every hostile emergency.”^130^

Gahagan's final point was that the hospitals were also a training ground for indigenous practitioners: “General Hospitals are the most useful and preparatory Asylums for Native Medical Servants, such as Assistant Apothecaries, Dressers, nursing attendants, and for various other purposes.”^131^

This highlights the other aspect of the search for distinction in the city of Madras: that from the region itself. The emergence of Madras as the principal site of medical innovation in the region was hardly a coincidence: it was the British stronghold on the coast. This particular metropolis/province confguration in scientific expertise was linked to commercial enrichment and displacement, a topic with a complex historiography. The relationship between metropolitan and provincial science in Europe has been well documented.documented.^132^ The links between science and trade have been explored, as well as those between commerce, commodities, and curiosities, and important arguments about how commerce has shaped the representation of nature have been formulated.been formulated.^133^ These have further enriched the already diverse literature on European history of science and medicine, particularly that of the eighteenth century. They have also contributed to our growing understanding of the peripheral contribution to the expansion of metropolitan knowledge systems.^134^

But what remains outside the scope of this historiography is the analysis[Other P-26] of the areas where provincial science originated. How were the shifts in knowledge and materials affected by changes in these “hinterlands”? This is where we have to turn to another major and perhaps equally diverse historiography, that of eighteenth-century India—particularly the debates on shifting patterns of trade and authority. The eighteenth century has occupied an important place in Indian historiography because it is often identifed as the period of transformation, of shifting authorities, cultures, and legitimacies. Historians have looked at it from different points of view: scholars like Irfan Habib and M. Athar Ali have seen it as a period of economic breakdown, subsequent to the decline of the Mughal Empire;^135^ others have linked the political decline to a decline in trade.^136^

Interestingly, here too networks have been traced: those connecting agrarian regions with the world economy in the centuries before 1800.^137^ Others have stressed that the overall relationship between European corporate enterprises and the Indian and other Asian authorities was often an amicable one, based on a perception of mutual advantage, which often led to an increase in trade and regional prosperity.^138^ Later studies have increasingly stressed the links between trade and the hinterland. Focusing particularly on the Coromandel Coast, Sinnapah Arasaratnam has shown the shifting fortunes of ports like Machilipatnam and Madras following increasing European participation.^139^ Others have analyzed these shifts more in terms of factors internal to Asian political systems.^140^ As Sanjay Subrahmanyam points out, the only really conspicuous feature of the early eighteenth-century Coromandel Coast was the expansion of the presence of the European country trader.^141^

What is obvious from a survey of the historiography of the eighteenth century is not a picture of a lack of dynamics or “vitality,” but one of shifting dynamics. Subrahmanyam captures the contours of this change.^142^ He analyzes how the European trading companies starved southern polities of important trade and tax revenues, weakening them and reducing regional prosperity, which in turn undermined the proftability of the companies. Thus, when Sa'adatullah Khan, the nawab of Arcot, attempted to establish a coastal trading center, he could not compete with the entrenched European trading enclaves that saturated the Coromandel Coast. The attempts of Haidar Ali and Tipu Sultan to drive out the EEIC and gain access to the coast also ended in failure: the Europeans had by then established themselves too securely. In the fourth Anglo-Maratha War, Tipu was killed and his territories subsequently annexed.

Thus the historiography of the commodifcation of curiosities, of trade and treatment, of commerce and science, and of material culture and knowledge, as developed for science in Europe, needs to be placed alongside another historiography of material culture: that describing the economic and political histories of shifting dynamics and sovereignties in areas beyond Europe. A link between the histories and historiographies of displacement and accretion thus needs to be forged. In the next section I explore these possibilities.

## Displaced Dynamics

By sending the Tanjavur physician along with his pills to Madras, Schwartz had confrmed that the Madras hospital by the 1780s, like the city in trade, had become the center of medicine along the Coromandel Coast. Similarly, Benjamin Heyne of the Tranquebar mission, a keen natural history enthusiast, had also migrated to the Madras establishment to pursue his scientifc projects.^143^ The “Tanjore pills” were to be accepted only after their “scientific” verification at Madras. The value of “experience,” vital in shaping the trajectory of scientifc practice in Madras, did not necessarily [Other P-28] supersede the value of trial, authentication, and certifcation. Gahagan, who failed Pasley's admission test of “experience and local knowledge” in Madras, could join the establishment when he returned in 1778 with his diploma from England.^144^ As Steven Shapin has shown, the credibility of scientifc claims was intertwined with issues of *trust,* which were shaped by the link between virtue and expertise.virtue and expertise.^145^ The journey of the pills, first from Tanjavur to Madras and then to London, was marked by the shifting centers of trust and authority.

Much of the centralization process in medical practice was prompted by the need of the military establishment to organize the medical establishment around Madras. In April 1760, for better integration among several hospitals in other parts of the Presidency, the Military Board resolved that one “General Hospital” was to be maintained in Madras, and the sick and the wounded at the garrisons at Arcot and Chinglepet were to be sent to Madras.^146^ Yet four General Hospitals continued to exist in the towns of Madras, Trichinopoly, Vellore, and Machilipatnam, and in 1786 with the formation of the Medical Department in Madras the question of centralization was raised again. Starting in 1764 medical services had been formed in the Presidencies (the Bengal Medical Service was formed in 1764, that of Madras in 1767, and that of Bombay in 1779) to regulate the appointment, succession, and wages of surgeons.^147^ In 1786 John Dalling, the commander-in-chief of Madras, expressed his unhappiness with the medical establishment, which had “loaded the Company with such an enormous expence.”^148^ He directed that the hospitals of district garrisons should be abolished, and the Presidency Hospital should become a general one for the sick and wounded from the regiments as well as for the recruits arriving from England. He also urged the formation of a Hospital Committee of Administration consisting of senior officers as surgeon general, a surgeon major, and two other surgeons.^149^

In April 1786 the Medical Department and the Hospital Board were formed, with James Anderson as the physician general.^150^ The Hospital Board was to recommend to the governor and the Council the promotion of surgeons; it was to prepare details regarding “Returns of Sick” and “Receipts of Medicine”; and it was to appoint a purveyor to take care of all the stores, except medicine, which was to be looked after by the apothecary.^151^ The Madras General Hospital was to be responsible for distributing all the medicines received from Europe among the four other hospitals.^152^ The organization was geared essentially toward military requirements, with no provision made for medical attendance on civil servants, who were to be supplied medicine from the dispensary of the fort. The ranks of the medical establishment were also decided according to the military hierarchy.^153^ Dalling defined clearly how Madras was to be the center of all the medical expertise of the Presidency:

Madras being the Capital of all the Hon'ble Company's possessions on the Coast, & where all persons coming from Europe, to serve under this Presidency, disembark,… it appears necessary to establish a General Hospital at this place for their immediate reception, in which the sick may receive every assistance that can be derived from the experience of the surgeons who from their long residence in this Country, & their knowledge of its productions, are best acquainted with the proper remedie to be used for the recovery of the Sick and also where such remedies are to be procured without delay.^154^

Militarization also implied the curbing of the role of the medical establishment. By the General Order dated 14 April 1802 the Medical Board was removed from nonmedical activities such as administration, including the right to reward or recommend someone for promotion or remuneration, and was to restrict itself to “those objects of Medical Practice, which belong to its immediate province.”^155^ All the decisions were henceforth to be made by the commander-in-chief. The General Hospitals of Trichinopoly, Vellore, and Machilipatnam were to be called “Garrison[Other P-30] Hospitals.” This was very different from the situation in England, where physicians of the Army Medical Board (formed in 1793) successfully asserted their independence—particularly regarding appointments and promotions—from the military establishment.^156^ The history of the medical establishment of Madras thus needs to be seen as one being increasingly subsumed within the growing requirements and infuence of the military establishment in the region. The successes of the military campaigns of the EEIC's army in the Deccan against the French, Mysore, and finally the Marathas had ensured vital territorial revenue, the “chief economic prize for the British in the East.”^157^

The medical establishment was opposed to such a military takeover. This is where the otherwise correlated processes of centralization and the search for autonomy came into confict. In response to these orders the Hospital Board expressed its “dismay” and “concern”: it cited the example of England, where the Medical Board recommended the appointments of medical staff, and it argued that the Medical Board had played a central role in running the entire medical establishment of the Presidency, a role that the Court of Directors had previously recognized.^158^ The Hospital Board was particularly keen on retaining its rights to decide about medical postings, rewards, and promotions; to that extent, autonomy from the military establishment was perceived as crucial:

With medical men therefore detached and living entirely in military Societies, cut off from the Head of their Department, and deprived of all hopes of success in the Service, by any attempts to recommend themselves, in the only proper way for Medical Men to do it is not difficult to foresee how much by such a system, the relish for professional pursuits in many young Practitioner must be lost, greatly to the future falling back on the Hon'ble Company's Medical Establishment and consequently to the prejudice and distress of the pubic service.^159^

Some boundaries, however, were put up by the surgeons themselves. The development of the Madras hospital as an important training site for indigenous practitioners was also a process in defining therapeutics. There were simultaneous efforts toward reducing the dependence on local practitioners, which sometimes refected an emerging distrust of them. This had interesting connotations in terms of both knowledge and politics.[Other P-31]

On the one hand, this was the period of Europe's assertion of its superior scientifc achievement vis-à-vis other regions, engendered in the duality discussed above. On the other hand, the Company was now establishing itself as the dominant regional power and reducing its dependence on local informants. This brings into question how far we can understand the interactions between the Europeans and Indians as “dialogues” and “conversations.” Scholars like Eugene Irschik, Thomas Trautmann, and Philip Wagoner, describing the nature of communication between the colonizer and the colonized in eighteenth- and nineteenth-century South India, have sought to assign a more “active” role to the colonized, to show that the “indigenous intellectuals in reality contributed actively to the process, and that colonial knowledge was thus produced through a complex form of collaboration between colonizers and colonized.”^160^ Their complex depictions fail, however, to address the multifaceted shifts in economic realities, trust, legitimacy, and agency underlining these “dialogues.”

The same year (1792) that the Hospital Board appointed Indian contractors for the supply of bazaar medicines, it also ordered that Indian “servants” (assistants or “black doctors”) were not to be allowed to prepare medicines or dress the wounded soldiers, for they were considered incompetent in these areas.^161^ Training also implied more specific roles for the local practitioners, often different from their traditional ones. In 1802, the Medical Board wrote to the Court of Directors that the “black doctors” attached to the Native Battalions should be dismissed “and such of them as may appear to be qualifed, be employed as Compounders and Dressers.”^162^ Thus, although by the end of 1786 a “native doctor” was attached to each Sepoy battalion, their roles and activities were clearly defined.^163^ The issue is not so much about straightforward enrichment, exclusion, or a soliloquial production of knowledge, as it is about shifting legitimacy.

The government, more than the medical establishment, had become suspicious of local assistants—even those trained by Europeans. In 1800, when the Medical Board wrote to the government about appointing a “Malabar Assistant” who had been “brought up” by a French chemist[Other P-32] at Pondicherry and who could help to produce compounds like lunar caustic and nitrous acid (which, they added, were expensive to get from England and came sparingly and often in damaged form),^164^ the Governor in Council refused to employ someone “who is but superficially skilled in Chymical preparations [and] instead of being imminently useful, might be the occasion of the most serious mischief.”^165^

The *dubashi* institution, “typified in the European enclave … [as] a creation of the eighteenth century,” is an important instance of this changing scenario in Madras.^166^ In the early decades of the century *dubashes* functioned as guides, advisors, brokers, and even moneylenders to the EEIC offcials. Their preeminence in the mid-eighteenth century over other local merchant groups indeed symbolized the ascendancy of Brahmin and Velalla^167^ power over traditional mercantile groups.^168^ But in late 1794, the Board of Revenue decided to make an all-out attempt to reduce the power of the Jagir Mirasidars^169^ and their Madras *dubashi* connections.^170^ By 1800, the *dubashi* infuence had almost withered away and the term disappeared from the official nomenclature.^171^ Part of the reason was that the Court of Directors now felt the *need* to dissociate themselves from these groups.^172^ The other was the *ability* of the EEIC to replace them: prohibitions regarding private trade reduced dependency on *dubashes* as agents, and linguistic training provided to junior civil servants in Madras reduced the need for translators or intermediaries.^173^

There were also limits to the surgeons' search for eminence and autonomy in medical research in Madras. To begin with, the status of the surgeons within the EEIC hierarchy was rather low. Financial rewards for[Other P-33] their services in difficult conditions were hardly forthcoming. In 1761, during a war with the French, when the Madras Military Board made a proposal for the increase of surgeons' salaries, it received a sharp admonition from the Court of Directors, the tone of which illustrates the standing of the colonial medical officers in the eyes of the authorities in London:

You tell us that the Salaries of our Surgeons must be enlarged if we expect or desire to have Men of Ability in their profession; The Surgeons that we send abroad to our Capital Settlements are always acquainted with their Salaries and Emoluments, and we find no difficulties in having able Men of that profession, as well as all other Branches of our Service, *if their heads there are turned, give us due Notice, that we may call them home again and supply their Places with Men of more humble minds, though perhaps not inferior Talents.*^174^

There was little infrastructure for the large-scale production of “Country” medicine to support the needs of the increasing army. Pasley responded to the wish of the Court of Directors that medicines be prepared in India by showing that such a plan was impractical due to the want of utensils, chemical laboratories, and trained assistants.^175^ Thus the dependence on European medicines continued, and in fact increased by the late eighteenth century: the Court of Directors noticed that the expenditure on European medicines had increased from £7,447 in 1798 to £12,994 in 1799, which the Board explained by saying that there was increased expenditure during the war with Tipu, and also that the native troops were being given more European medicines.^176^ The trend continued, and in 1802 the Medical Board had once more to justify the increased use of European medicines in terms of increased military size, veterinary requirements, and the fact of “the Native Troops being now as regularly attended and prescribed for by the surgeons as the European Troops are, which was not the case formerly, and has been gradually effected.”^177^ New medicines, particularly chemicals developed in Europe, were also regularly put on trial here,^178^ and the reorganization of the Medical Board was followed[Other P-34] by intense smallpox vaccination programs from 1800 within and outside the army establishment in Madras.^179^

The history of the growing prosperity of the Society of Apothecaries in London is related to these developments in Madras. In 1766 the Society obtained the monopoly for supplying medicine to the EEIC. This became the core of its business, and a period of flourishing profits in the late eighteenth century followed. In the 1820s it was the chief supplier of the huge Indian army of approximately 300,000 men. The Society boasted: “the whole army of India (we believe exclusively) is served under the direction of the Hon United India Company from our establishment.”^180^ The Navy Stock of the Society yielded an average of £20,160 a year for the first decade of the nineteenth century,^181^ and throughout most of the nineteenth century, trade and the Navy Stock were the mainstays of the Society's activities and prosperity. In fact, this increasing focus on revenue and trade by the Society came in for criticism.^182^ The shifting dynamics and fortunes of medicine had thus followed those of trade.

One incident serves to illustrate the nature of medical practice in Madras at the end of the century. In 1800 Thomas Evans, who had very recently joined the Madras establishment as an assistant surgeon, made a proposal for the establishment of a dispensary in the Black Town. Evans had two justifications: first, such a dispensary would help to promote European medicine in these parts, thereby making commercial proft for the EEIC; second, it would help to suppress all “irregular practitioners and vendors of medicine in Black Town.”^183^ The Medical Board, however, rejected the plan and suggested that the surgeon posted in the Black Town and jail should check the “spurious” practitioners; a dispensary whose purpose would be to “dispose of as much medicine wholesale or retail as possible” would have, according to them, little effect to that regard.^184^ The government, on the other hand, recognized the merits of Evans's plans.[Other P-35] On 4 November the Governor in Council resolved that such a dispensary was to be established, which “would not only relieve the Company from an immediate expence, *but may ultimately increase the demand for European Medicines to an object of commercial importance.”* ^185^ They opined that the dispensary would also help to stop the “clandestine sale of medicine” and other “spurious medical practices” and to “introduce into India the knowledge of European Pharmacy, to remove by degrees the errors of ignorance and to extend the practice of scientific adepts.”^186^

By the middle of the nineteenth century, bazaar medicine had come a long way in its metropolitan participation. Edward John Waring (a surgeon in the Madras Medical Service, and physician to the rajah of Travancore), in his celebrated *Remarks on the Uses of Some of the Bazaar Medicines and Common Medical Plants of India,* written from England, expressed the need to “introduce” bazaar medicine to these people in the colony: to the missionaries working there, the resident Europeans and Anglo-Indians, and the Indians, “the daily increasing array of educated Natives, who as a class, readily avail themselves of every scrap of knowledge drawn from trustworthy European sources, which tends to throw light on the products and resources of their native land.”^187^ The line between Country and European medicine, much like that between Country and European trade, had indeed become blurred.

## Conclusion

Eighteenth-century Madras was a site where both “Western” and “Eastern” medicines were subjected to trial. War, isolation, cultural exchanges, and climate had expanded and redefined the possibilities of these practices. On the other hand, networks, collaborations, and negotiations were vital to European endurance in these distant lands, and trade was both the means and the metaphor of these exchanges. The “rise” of the bazaar from the eighteenth century has been seen as a new “distinct world” of sophisticated negotiation and bargaining developed by the indigenous[Other P-36] trading communities in the wake of European imperialism.^188^ In this paper I have stressed the need to understand the nature of these negotiations and accumulations against the backdrop of trade and dominance.

Since it was not just medicine that was being bartered here, the general economic history of Coromandel trade becomes relevant. A comparative analysis of Britain and South India in the eighteenth century in terms of wages, prices of grain, and agricultural productivity shows that the image of “impoverished masses” of India, as painted by European travelers, was misleading. Such an analysis has not only challenged concepts of Oriental despotism and static and self-sufficient villages, but also has hinted at the need to rethink Europe's industrial revolution, which could be seen as a response to the “economic dynamism outside,” Britain's answer to India's competitive challenge.^189^

On the other hand, as one of the largest employers in eighteenth-century Britain, the EEIC shaped British middle-class earning and spending. The EEIC clerks were more able to earn higher salaries, compared with other banking, commercial, and governmental employees between 1750 and 1850, and the economy generally benefted from vigorously escalating salaries in these classic years of industrial revolution, which in turn led to domestic demand for the goods of the new industrial sector.^190^ There were of course the accumulated credits from India, which helped to finance Britain's land warfare with the French.^191^

It is necessary to align the history of medicine with economic history: not only to write an economic history of medicine, but also to align the trajectories of intellectual and material wealth. These triumphs of the eighteenth century contributed to the power of nineteenth-century Western science otherwise visible in its identifcation of microbes and vectors and in its eradicative and preventive successes in the tropics. This complex world of Madras—its *dubashes,* “black doctors,” surgeons, and arsenics, its shifting dynamics and sovereignties, and its tributes of dried seeds and texts sent to Europe—can thus inform us more about the researches and resources of Western science.
